# Practical Points

**Published:** 1904-03-12

**Authors:** 


### PRACTICAL POINTS.

#### SUICIDES AND HOSPITALS.

THE HON. SECRETARY of the Wantage Cottage Hospital writes: Would you be good enough to inform me if it is the practice for Cottage Hospitals without special accommodation to receive persons who have attempted to commit suicide?

*** It is a common practice for the police to take cases of suicide, where the patient is suffering from injuries, whether self-inflicted or not, or from poison, to the hospital, and for the hospital authorities to admit them when the cases are such as to necessitate hospital care. By arrangement with the police, or otherwise, such cases may be in charge of the police during the whole period of their residence.—ED. THE HOSPITAL.

